# Immune Profiling of Peripheral Blood Mononuclear Cells at Pancreas Acute Rejection Episodes in Kidney-Pancreas Transplant Recipients

**DOI:** 10.3389/ti.2022.10639

**Published:** 2022-11-18

**Authors:** Jordi Rovira, Maria Jose Ramirez-Bajo, Elisenda Bañón-Maneus, Natalia Hierro-Garcia, Marta Lazo-Rodriguez, Gaston J. Piñeiro, Enrique Montagud-Marrahi, David Cucchiari, Ignacio Revuelta, Miriam Cuatrecasas, Josep M. Campistol, Maria Jose Ricart, Fritz Diekmann, Angeles Garcia-Criado, Pedro Ventura-Aguiar

**Affiliations:** ^1^ Laboratori Experimental de Nefrologia i Trasplantament (LENIT), Institut d’Investigacions Biomèdiques August Pi i Sunyer (IDIBAPS), Barcelona, Spain; ^2^ Red de Investigación Renal (REDinREN), Madrid, Spain; ^3^ Renal Transplant Unit, Nephrology and Kidney Transplant Department, Hospital Clinic de Barcelona, Barcelona, Spain; ^4^ Pathology Department, Center for Biomedical Diagnosis, Hospital Clinic de Barcelona, Barcelona, Spain; ^5^ Radiology Department, Center for Imaging Diagnosis, Hospital Clinic de Barcelona, Barcelona, Spain

**Keywords:** graft rejection, simultaneous pancreas kidney transplantation, immune profiling, peripheral blood mononuclear cells, T cells, B cells

## Abstract

Profiling of circulating immune cells provides valuable insight to the pathophysiology of acute rejection in organ transplantation. Herein we characterized the peripheral blood mononuclear cells in simultaneous kidney-pancreas transplant recipients. We conducted a retrospective analysis in a biopsy-matched cohort (*n* = 67) and compared patients with biopsy proven acute rejection (BPAR; 41%) to those without rejection (No-AR). We observed that CD3+ T cells, both CD8+ and CD4+, as well as CD19+ B cells were increased in patients with BPAR, particularly in biopsies performed in the early post-transplant period (<3 months). During this period immune subsets presented a good discriminative ability (CD4+ AUC 0.79; CD8+ AUC 0.80; B cells AUC 0.86; *p* < 0.05) and outperformed lipase (AUC 0.62; *p* = 0.12) for the diagnosis of acute rejection. We further evaluated whether this could be explained by differences in frequencies prior to transplantation. Patients presenting with early post-transplant rejection (<3 months) had a significant increase in T-cell frequencies pre-transplant, both CD4+ T cells and CD8+ T cells (*p* < 0.01), which were associated with a significant inferior rejection-free graft survival. T cell frequencies in peripheral blood correlated with pancreas acute rejection episodes, and variations prior to transplantation were associated with pancreas early acute rejection.

## Graphical Abstract

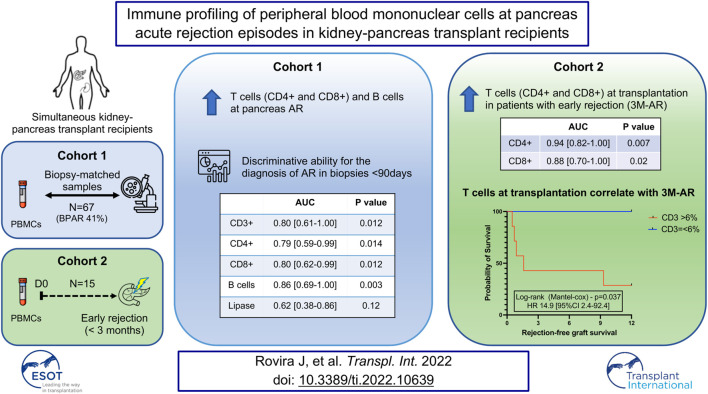

## Introduction

Immune profiling in solid organ transplant recipients has contributed to an increase in the understanding of the pathophysiology of acute rejection (1). This approach has also lead to novel insights in other solid organs transplants, such as kidney (2–5), liver (6), heart (7), and lung transplantation (8, 9). There have been several studies highlighting the relevance of subsets of T cells and B cells on the outcome of organ transplantation (4, 5, 10, 11). This understanding of transplant immunology has led to the development of strategies to mitigate immunosuppression side effects, by identification of donor-specific B and T cells prior to transplantation and adjusting immunosuppression accordingly (12), or through the treatment with regulatory cell therapies (13).

Immune profiling and functional characterization of peripheral blood mononuclear cells (PBMCs) in kidney transplant recipients (KTRs) provides relevant information regarding induced immunosuppressive status (14), and immunological risk (2, 15, 16, 17), and correlate with long-term graft survival (3). Despite the simplicity of PBMCs phenotyping compared to functional (12, 18), and genetic analysis (19, 20), their characterization have been crucial to unravel the pathophysiology of rejection and tolerance (21, 22). We now know that regulatory T (Tregs) (17) and B cells (Bregs) (23) are both increased in immune tolerant patients, and that T (17), B (19), and NK (24) cell subsets in peripheral blood correlate with graft acute rejection.

Simultaneous Pancreas Kidney transplantation (SPKTx) is the best treatment alternative for patients with insulin-dependent diabetes mellitus (DM) and end stage renal disease (ESRD) (25, 26). Pancreas graft rejection remains as the leading cause of graft failure after the first 90 days, with acute rejection incidences up to 21% in the first 12 months (27–31). Several risk factors for acute rejection have been identified, such as are donor age, pancreas cold ischemia time, donor cause of death (32), transplantation type (29, 30), and the presence of donor specific antibodies (DSA) (33–35). Despite a high incidence of acute rejection, peripheral blood immune profiling during acute rejection episodes is scarce.

Herein we present a study aiming at characterizing circulating leukocytes in recipients of simultaneous pancreas-kidney transplantation. The main objectives were to explore the differential expression of circulating leukocytes during episodes of pancreas acute rejection, and to explore the correlation between the pre-transplant rates of different leukocytes subsets and the development of acute rejection in the early post-transplant period.

## Materials and Methods

### Study Design and Patient’s Population

Peripheral blood mononuclear cells (PBMCs) were collected from pancreas transplant recipients admitted for pancreas transplantation and at the time of pancreas graft biopsies. Biopsies were performed either for-cause or for surveillance. Blood collection was performed prior to the transplant or the biopsy procedure. For this study we conducted a retrospective analysis using the stored patient samples. Collection and use of patient blood samples for the current study was approved by local ethical IRB board (HCB_2016_0479) and was conducted in full adherence to the Declaration of Helsinki. All patients provided written informed consent to participate in the study.

Between January 2017 and December 2019 a total of 108 pancreas graft biopsies were performed in our center. We excluded all samples in which a biopsy-matched blood sample was not obtained (*n* = 17), and those in which graft biopsy could not be performed or sample was not suitable for histological diagnosis (*n* = 24). During the same period a total of 15 simultaneous pancreas-kidney transplants were performed, from which PBMCs were available both at 1) the day of transplantation; and 2) pancreas graft biopsy performed within the first 3 months (either for surveillance or for cause, whichever was first).

### Pancreas Graft Biopsies and Blood Samples

For cause biopsies were indicated according to hospital´s protocol–1) >3xs increase in serum amylase or lipase; 2) hyperglycemia (fasting blood glucose >120 mg/dl); 3) *de novo* donor-specific antibodies (DSA); or 4) *de novo* anti-glutamic acid decarboxylase antibodies (GAD). Surveillance biopsies were performed according to center protocol at 3 weeks and at 12 months after transplantation, or as surveillance 4 weeks following the completion of the treatment for an acute rejection episode. Samples were obtained by ultrasound-guided percutaneous needle punch. Histological and immunohistochemical evaluation of pancreas graft biopsies was performed according to the 2011 Banff criteria (36, 37).

Blood samples were obtained contemporaneously to pancreas graft biopsy and used to measure glucose (mg/dl), amylase (U/L), lipase (U/L), creatinine (mg/dl), C-Peptide (ng/ml), HbA1C (%), and anti-GAD (U/ml). Serum samples at time of biopsy were screened for HLA class I and II antibodies using the Lifecodes LifeScreen Deluxe flow bead assay (Immucor, Stamford, CT, United States). Antibody specificities, including the presence of DSA, were determined using the Lifecodes Single Antigen bead assay (Immucor, Stamford, CT, United States) in patients with positive screening for HLA antibodies.

### Characterization of Circulating Leukocytes

Blood samples were collected in two separate EDTA tubes and processed separately. In one red blood cells (RBCs) were removed from whole blood samples with RBC lysis buffer (Invitrogen™) and cells were resuspended at a concentration of 10^6^/ml in complete MACS buffer to determine a broad spectrum of leukocytes. In the other, PBMCs were isolated from whole blood samples by standard Ficoll density gradient (Ficoll-Paque premium, GE healthcare Bio-Science AB) and were resuspended at a concentration of 10^6^/ml in complete MACS buffer to determine T and B cell subsets. Six different panels were designed aiming at interrogating the immune cells for markers of cell activity, memory, and differentiation, with focus on T and B cells. The gating of T cell subsets and B cell subsets are defined in Supplementary Figure S1. Cell surface markers were stained with antibodies indicated in Supplementary Table S1, and used according to the instructions of the manufacturer. Except for the leukocyte panel, Aqua Live/Dead fixable dead cell kit (Thermo Fisher Scientific, Waltham, MA, United States) was used unambiguously to remove dead cells. Flow cytometry analysis was performed on a FACS Canto II (BD Biosciences, Heidelberg, Germany). Data were analyzed using FlowJo software (Tree Star, Ashland, OR, United States).

### Immunosuppression Protocol

Induction therapy was used in all patients with rabbit anti-human lymphocytes polyclonal antibodies (Thymoglobulin 1.25 mg/kg/d for 4 consecutive days), and maintenance immunosuppression protocol with tacrolimus, mycophenolate, and prednisone. Prednisone withdrawal was attempted between months 3–6 in all patients with low immunological risk, absence of acute rejection episodes during the first 90 days, and good tolerance to mycophenolate treatment doses.

### Statistical Analysis

Comparisons of median measurements were performed using Mann-Whitney U test and *p* value < 0.05 was considered statistically significant. Kaplan–Meier was used to estimate unadjusted patient, graft, and rejection-free survivals and compared using log-rank test. Binominal logistic regression was used to calculate odds ratio, and Cox proportional regression performed to estimate grafts’ hazards. Statistical analysis was performed using SPSS (version 22, IBM, United States) software, with all tests 2-tailed and significance considered if *p* < 0.05.

## Results

### Immune Profile in the Peripheral Blood at Pancreas Acute Rejection

A total of 67 biopsy-matched (biopsy-proven acute rejection [BPAR] *n* = 28; No rejection *n* = 39) PBMCs samples were performed during the period analyzed. Most biopsies were performed per protocol (52%) or surveillance following treatment of an acute rejection (AR) episode (23%), and performed for-cause in 18 cases (25%), with a median time from transplant to biopsy of 11.9 months [IQR 0.9–13.8]. At time of biopsy 18 patients (28%) presented DSA. Patients with a BPAR had higher lipase (*p* < 0.001) and glucose levels (*p* = 0.02), without differences in serum creatinine nor amylase, compared to those without AR. The demographics and immunological parameters at time of biopsy are described in detail in Table 1.

**TABLE 1:** Patients’ demographics in biopsy-related samples.

	Overall (*n* = 67)	No-AR (*n* = 39)	BPAR (*n* = 28)	P
Age at biopsy (years)	40.9 ± 9.7	40.5 ± 8.7	41.4 ± 11.1	0.75
Gender (male;%)	55%	51%	61%	0.30
Type of Transplant				0.27
SPK (%)	87%	85%	89%	
PAK (%)	13%	15%	11%	
Indication for biopsy (n[%])				0.053
For-cause	17 (25%)	10 (26%)	7 (25%)	
Surveillance post-rejection	15 (23%)	5 (13%)	10 (36%)	
Per protocol 3 weeks	18 (27%)	11 (28%)	7 (25%)	
Per protocol 12 months	17 (25%)	13(33%)	4 (14%)	
Time to biopsy (months)	11.9 [0.9–13.8]	12 [0.9–13.3]	11.5 [1.2–19.2]	0.37
cPRA (%)				
Class I	0 [0–16]	0 [0–11]		0.81
Class II	16 [0–51]	0 [0–46]		0.49
Total	45 [0–54]	22 [0–60]		0.99
DSA (yes; n [%])	19 (28%)	8 (21%)	11 (38%)	0.40
*De novo* (% of DSA+)	92%	75%	100%	
Amylase (U/L)	99 [73–145]	100 [73–140]	98 [73–166]	0.51
Lipase (U/L)	45 [30–82]	38 [24–63]	69 [49–144]	<0.001
Glucose (mg/ml)	89 [79–108]	85 [76–96]	93 [81–116]	0.023
HbA1C (%)	5.6 ± 1.6	5.7 ± 1.5	5.4 ± 1.7	0.90
C-Peptide (ng/ml)	3.3 [2.3–4.6]	3.5 [2.4–4.9]	2.8 [2.1–4.6]	0.26
Anti-GAD (U/mL)	0.3 [0.1–2.7]	0.3 [0.1–3.4]	0.2 [0.1–2.6]	0.86
sCreatinine (mg/dl)	1.29 ± 0.6	1.40 ± 0.7	1.14 ± 0.4	0.23
eGFR (ml/min/1.73 m^2^)	70 ± 24	66 ± 27	76 ± 20	0.15
Immunosuppression				
Prednisone	94%	92%	97%	0.15
Tacrolimus	97%	97%	96%	0.64
Mycophenolate	94%	95%	93%	0.67
Sirolimus	8%	5%	11%	0.46
Banff Category				
No rejection	58%			
Indeterminate	10.4%		*n* = 7	
Acute Cellular grade 1	20.9%		*n* = 14	
Acute cellular grade 2	7.5%		*n* = 5	
Acute cellular grade 3	1.5%		*n* = 1	
Antibody mediated rejection	1.5%		*n* = 1	

**FIGURE 1:**
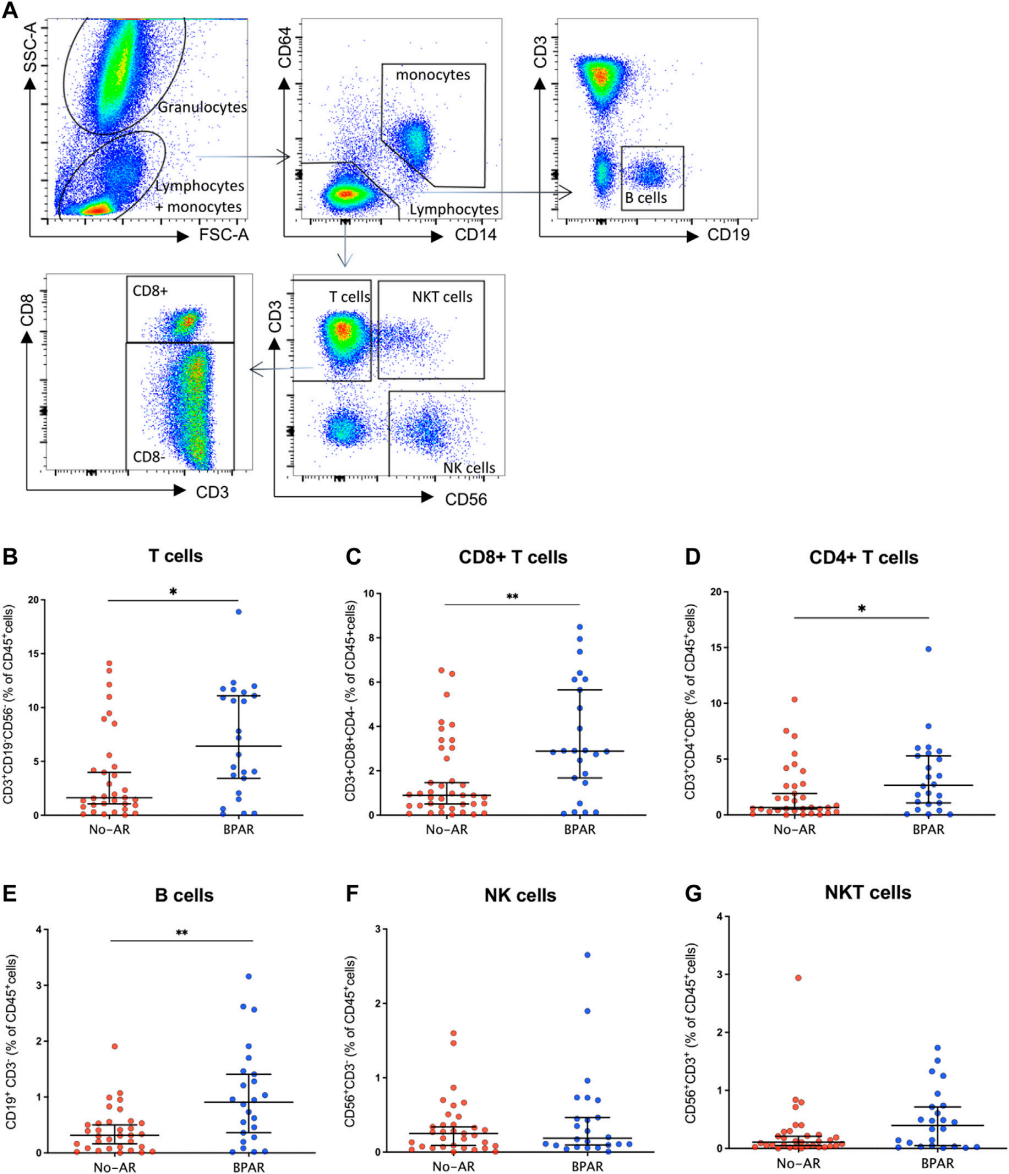
Immune cell lineages during acute rejection episodes. Gating strategy for the characterization of circulating leukocytes **(A)**. CD3^+^ **(B)** CD8^+^ **(C)**, and CD4^+^ **(D)** T cells; B cells **(E)**, NK cells **(F)**, and NKT cells **(G)** have been presented as percentages of total CD45^+^ cells in *n* = 28 biopsy-matched blood samples with acute rejection and *n* = 39 without rejection. Box plots were calculated using unpaired Mann–Whitney U test. *p < 0.05; **p < 0.01; ***p < 0.001. Mean with SEM.

**FIGURE 2:**
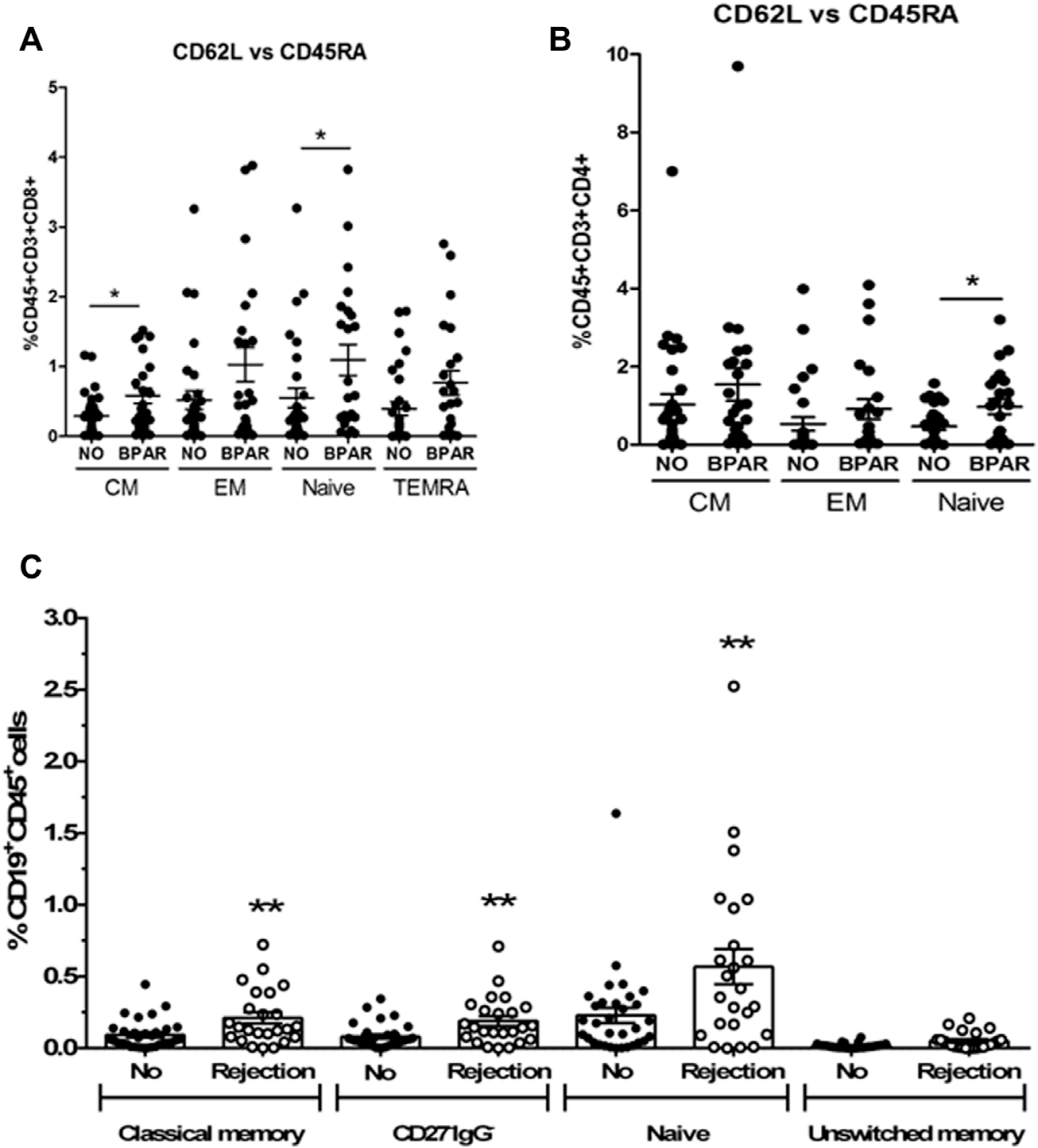
T and B cell subsets during acute rejection episodes. Relative frequencies of CD8^+^ T cell subsets **(A)**: CD8 Central memory (CM, CD3^+^CD8^+^CD45RA^−^CD62L^+^), CD8 Effector memory (EM, CD3^+^CD8^+^CD45RA^−^CD62L^−^), CD8 Naive (CD3^+^CD8^+^CD45RA^+^CD62L^+^), CD8 terminal differentiated effector memory (TEMRA, CD3^+^CD8^+^CD45RA^+^CD62L^−^). Relative frequencies of CD4^+^ T cell subsets **(B)**: CD4 Central memory (CM, CD3^+^CD4^+^CD45RA^−^CD62L^+^), CD4 Effector memory (EM, CD3^+^CD4^+^CD45RA^−^CD62L^−^), CD4 Naive (CD3^+^CD4^+^CD45RA^+^CD62L^+^). Relative frequencies of B cell subsets **(C)**: naïve (CD27^−^IgD^+^), unswitched memory (CD27^+^IgD^+^), classic memory (CD27^+^IgD^−^) and double negative CD27^−^IgG^−^ cells. Biopsy-matched blood samples from patients with a biopsy proven acute rejection (BPAR) has been compared to those without rejection (No AR). Box plots were calculated using unpaired Mann–Whitney U test. *p < 0.05; **p < 0.01. Mean with SEM.

Phenotypic characterization of immune cells by flow cytometry showed a significant increase of T cells (CD3^+^CD19^−^CD56^−^) (*p* = 0.0187) in BPAR compared to those without AR (Figures 1A,B), including CD8^+^ (CD3^+^CD8^+^CD4^−^; *p* = 0.007) and CD4^+^ (CD3^+^CD4^+^CD8^−^; *p* = 0.0459) T cell lineages (Figures 1C,D). The only T cell subsets significantly increased in patients with BPAR were CD8^+^ naïve and central memory (Figure 2A), and CD4^+^ naïve (Figure 2B). No other major differences within the CD8^+^ or CD4^+^ lineages were observed between groups, neither TCRαβ^+^ nor TCRγδ^+^ (*p* > 0.05). The percentage of B cells (CD19^+^CD3^−^) was higher in BPAR group compared to biopsies without signs of rejection (*p* = 0.005) (Figure 1E). A deeper analysis of B cell subsets using anti-CD27 and anti-IgD antibodies revealed that both naive and classical memory B cells were increased in patients with BPAR (Figure 2C; Supplementary Figure S1B). In addition, the percentages of NK (CD3^−^CD56^+^), of NKT (CD3^+^CD56^+^) and monocytes (CD14^+^) were not different between those with or without BPAR (Figures 1F,G).

### CD4^+^ and CD8^+^ T Cells Discriminate Early Acute Rejection

**FIGURE 3:**
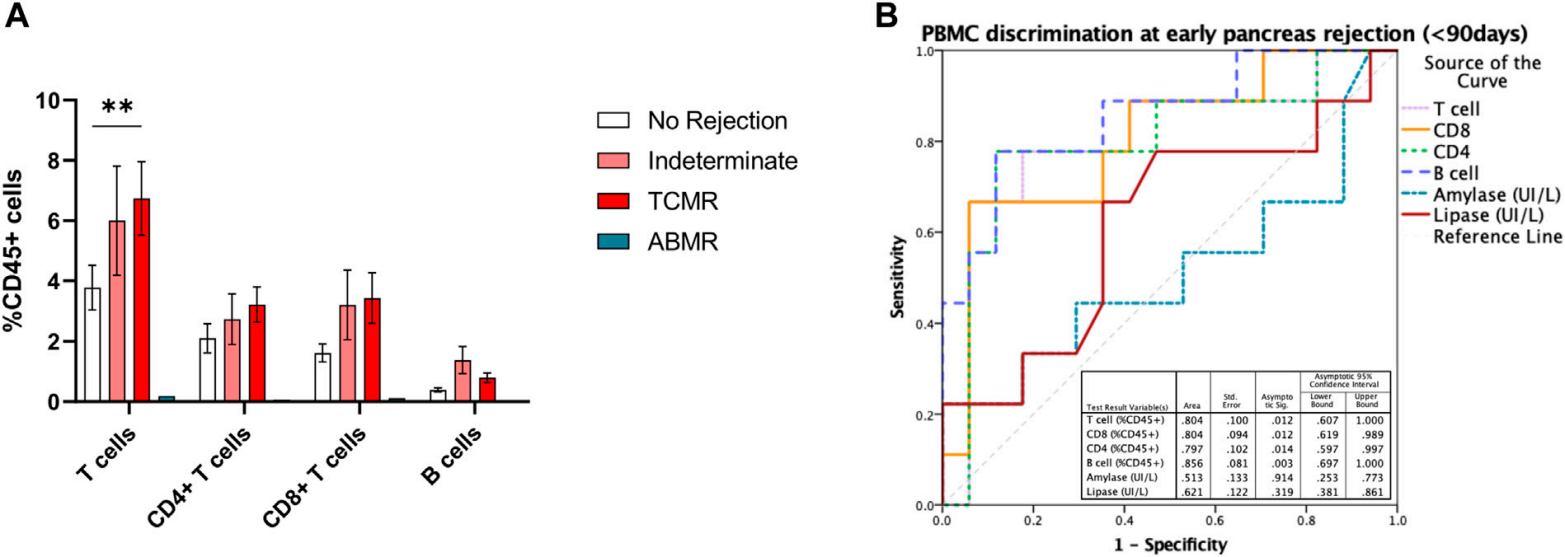
T and B cell subsets in early pancreas graft rejection. Relative frequencies of CD19^+^ B cells, CD3^+^ T cells, and its subsets CD4^+^ and CD8^+^ T cells **(A)** in patients with biopsies performed during the first 3 months after pancreas transplantation, and correlation with histological classification. Receiver operating curve (ROC) performed to discriminate the ability of B and T cell subsets in peripheral blood to discriminate pancreas acute rejection in the first 3 months after pancreas transplantation **(B)**. Box plots were calculated using unpaired Mann–Whitney U test. *p < 0.05; **p < 0.01; ***p < 0.001. Mean with SEM.

Having identified that both CD8^+^ and CD4^+^ T cells, as well as B cells, were increased at the time of acute rejection, we explored their ability to classify between those with and without BPAR. In a receiver operating curve analysis, both CD4^+^ (AUC 0.66 [95% CI 0.53–0.80]; *p* = 0.027), CD8^+^ (AUC 0.68 [95% CI 0.54–0.82]; *p* = 0.017), and B cells (AUC 0.69 [95% CI 0.55–0.83]; *p* = 0.012) presented a poor discriminative capacity. Most relevant, lipase outperformed any of the cell markers (AUC 0.72 [95% CI 0.58–0.85]; *p* = 0.004). We then evaluated whether timing post-transplant could influence the correlation between immune cell subtypes and acute rejection episodes. We observed that in biopsies performed in the early post-transplant period (<90 days) CD3^+^ T cells were increased in patients with T cell mediated rejection compared to those without rejection (Figure 3A), with a tendency towards an increase also in patients with indeterminate for rejection. Within this period T cells (AUC 0.80 [95% CI 0.61–1.0]; *p* = 0.012), both CD4^+^ (AUC 0.79 [95% CI 0.59–0.99]; *p* = 0.014) and CD8^+^ (AUC 0.80 [95% CI 0.62–0.99]; *p* = 0.012), and B cells (AUC 0.86 [95% CI 0.69–1.0]; *p* = 0.003) outperformed lipase for the diagnosis of acute rejection (AUC 0.62 [95% CI 0.38–0.86]; *p* = 0.12) (Figure 3B).

### Concomitant Kidney Graft Rejection

Since patients included in the analysis were recipients of simultaneous kidney-pancreas transplantation, we assessed whether there were concomitant kidney graft rejections at the time points evaluated, either concordant with pancreas graft rejection in BPAR group, or discordant in the no rejection group. Since per hospital policy simultaneous biopsies to both organs are not performed, we observed that in only one case there was a concomitant (on consecutive days) kidney graft biopsy, which was concordant with severe T cell mediated rejection - grade IIA in the kidney and grade 3 in the pancreas graft. Though serum creatinine is not a sensitive marker for kidney graft rejection, in particular subclinical rejection, we explored whether there were unperceived differences between groups. Both groups presented similar serum creatinine (BPAR 1.14 ± 0.71 vs. no AR 1.40 ± 0.37 mg/dl) at time of pancreas graft biopsy (Table 1). Moreover, no differences between groups were observed when patients were stratified by indication for and histological classification of pancreas graft biopsy (for cause vs. surveillance; BPAR vs. no rejection; *p* > 0.05).

### Immune’ Profiling at Pancreas Transplantation

Having identified that the discriminative ability of T cells in peripheral blood was particularly pronounced in the early post-transplant period, we explored whether these could be associated with an increased relative number prior to transplantation, or a resistance to T-cell depleting therapy. To do so we performed the immune profile from 15 patients who received a first simultaneous pancreas-kidney transplantation (SPK) and had a biopsy performed during the first 3 months after transplantation. Five had BPAR (3M-AR), whereas the remaining 10 had normal graft biopsies (No-AR).

**FIGURE 4:**
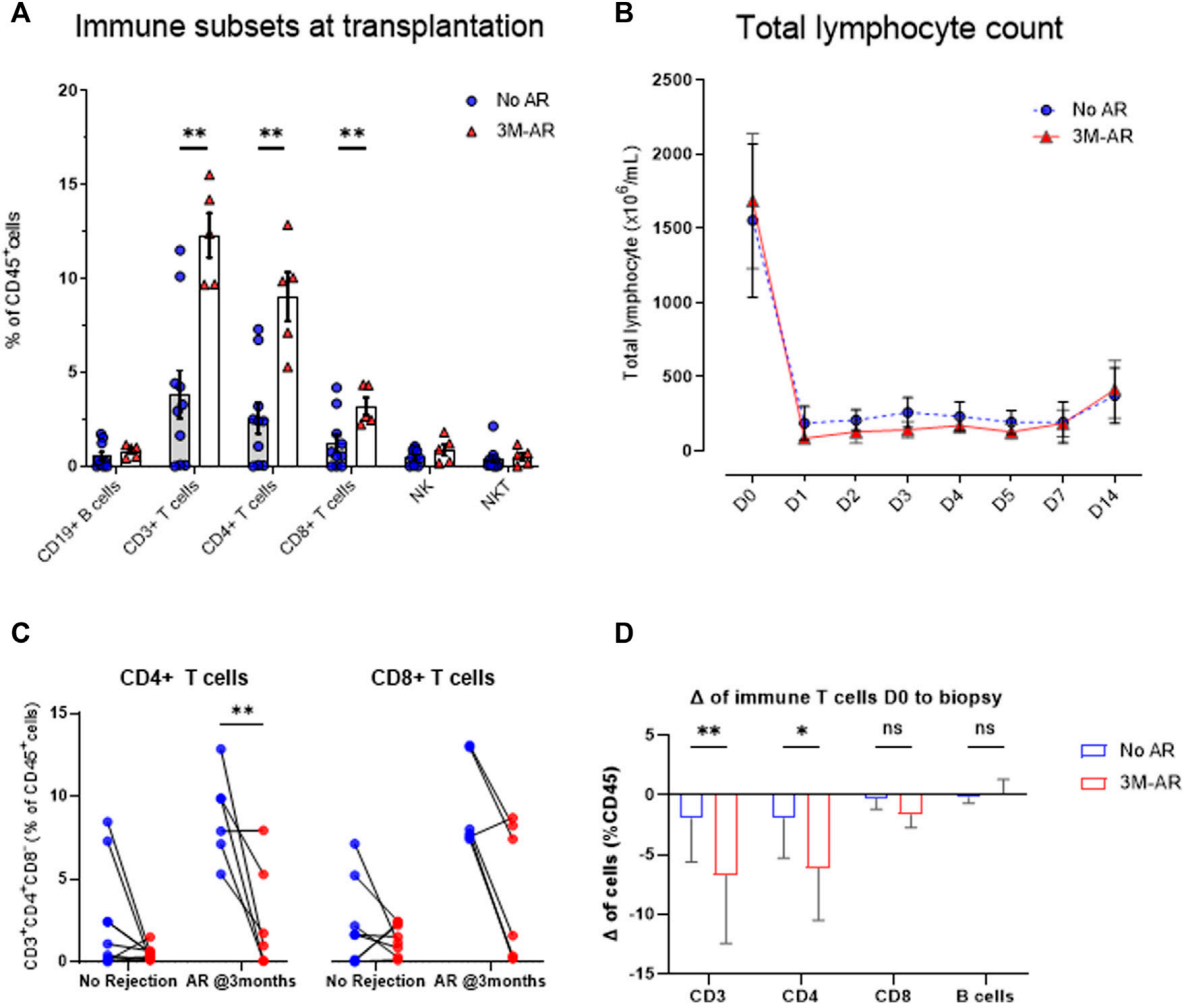
Immune cell lineages at time of transplantation. At time of transplantation, patients who eventually developed an acute rejection episode during the first 3 months post-transplant (3M-AR) presented a higher rate of CD3^+^, CD8^+^, and CD4^+^ T cells (*p* < 0.01) compared to those without rejection (No AR). No differences were observed on B cells, nor NK cells **(A)**. Significant decrease of total lymphocyte count in whole blood during the first days due to the use of T -cell depleting antibodies **(B)**, stratified by those who presented an acute rejection episode during the 3 months (3M-AR) compared to those without rejection (no AR) proven by biopsy. Delta in peripheral blood CD4^+^ and CD8^+^ T cell frequencies from the time of transplant to the day of first biopsy, stratified by presence (3M-AR) or absence (No AR) of acute rejection–individual patient data **(C)** and average of cohort **(D)**. Box plots were calculated using unpaired Mann–Whitney U test. *p < 0.05; **p < 0.01; ***p < 0.001. Mean with SEM.

**FIGURE 5:**
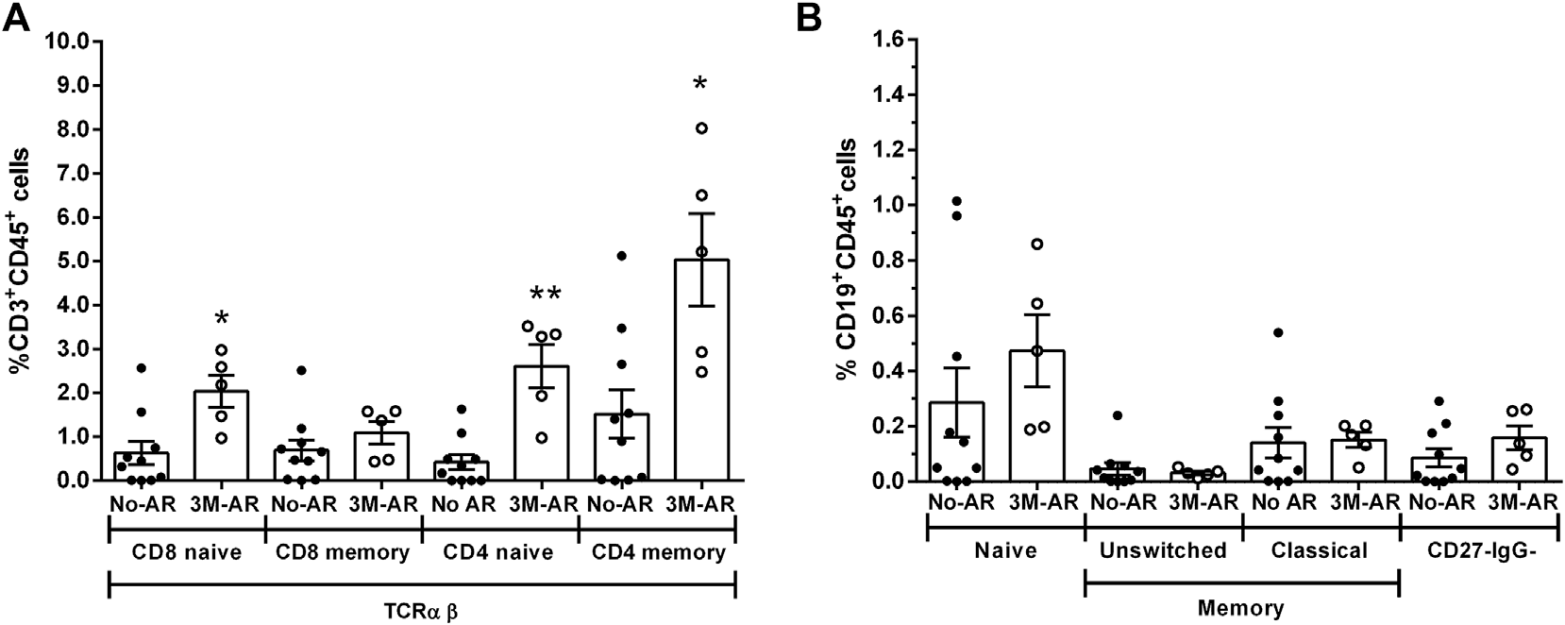
T and B cell subsets at time of transplantation. Relative frequencies of T cell subsets in TCRαβ **(A)**: CD8 naïve (CD3^+^CD8^+^CD45RA^+^CDRO^−^), CD8 memory (CD3^+^CD8^+^CD45RA^−^CDRO^+^), CD4 naïve (CD3^+^CD8^+^CD45RA^+^CD45RO^−^), CD4 memory (CD3^+^CD8^+^CD45RA^−^CD45RO^+^) cells. Relative frequencies of B cell subsets **(B)**: naïve (CD27^−^IgD^+^), unswitched memory (CD27^+^IgD^+^), classic memory (CD27^+^IgD^−^) and double negative CD27^−^IgG^-^ cells. Blood samples from patients at time of transplantation who presented a biopsy proven acute rejection during the first 3 months (3M-AR) has been compared to those without rejection during the same period (No AR). Box plots were calculated using unpaired Mann–Whitney U test. *p < 0.05; **p < 0.01. Mean with SEM.

At the time of transplantation, 3M-AR patients showed a significant increase of T cell populations (CD3^+^, CD8^+^ and CD4^+^; *p* < 0.005) (Figure 4A). However, the percentage of B and NK cells were comparable in both groups (Figure 4A). More detailed analyzes on T and B cell subsets using surface markers revealed that 3M-AR patients had higher levels of TCRαβ^+^ naive T cells, either CD4^+^ and CD8^+^ cells, and memory CD4^+^ T cells compared to patients without rejection (Figure 5).

To evaluate the response to T-cell depleting therapy, thymoglobulin, we firstly analyzed the total lymphocyte depletion in peripheral blood during the first days after transplantation. The reduction and subsequent lymphocyte recovery was similar between both groups (Figure 4B) during the first 14 days after transplant. Nonetheless, at time of first biopsy (BPAR median 0.7 months, no rejection median 1.2 months; *p* = 0.12), the reduction in T cells, particularly in CD8^+^ T cells, was higher in those with BPAR compared to no rejection group (Figures 4C,D).

### T Cells at Transplantation Correlate With Early Acute Rejection

**FIGURE 6:**
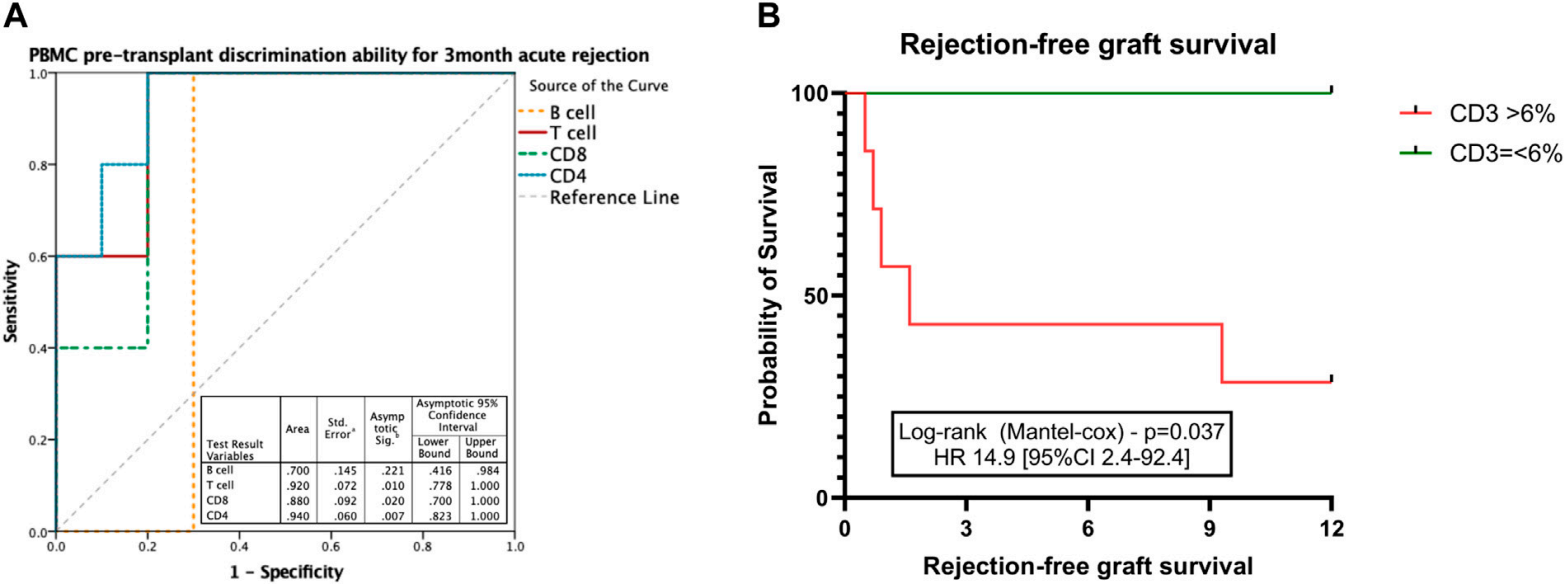
Discriminative ability for early acute rejection of T cell subsets at time of transplantation. ROC curve performed to discriminate the ability of CD3^+^ T cell count at transplantation to correlate with pancreas acute rejection at 3 months after transplantation **(A)**. Kaplan-Meier estimated pancreas rejection-free graft survival according to CD3^+^ T cell frequencies at the time of transplantation **(B)**. Box plots were calculated using unpaired Mann–Whitney U test. *p < 0.05; **p < 0.01; ***p < 0.001. Mean with SEM.

We then explored whether immune profiling at pancreas transplantation (D0) could correlate with the risk of acute rejection early after transplantation. Having identified that in peripheral blood both CD8^+^ and CD4^+^ T cells, as well as B cells, were increased during early acute rejection episodes, we explored their ability to classify those at risk for early acute rejection prior to transplantation. In a receiver operating curve analysis, both CD4^+^ (AUC 0.94 [95% CI 0.82–1.0]; *p* = 0.007) and CD8^+^ (AUC 0.88 [95% CI 0.70–1.0]; *p* = 0.020) presented good discriminative capacity, whereas B cells failed (AUC 0.70 [95% CI 0.42–0.98]; *p* = 0.221) (Figure 6A). At cut-off of 6%, CD3^+^ T cells presented a sensitivity of 100% and a specificity of 70% for the diagnosis of acute rejection. We then stratified patients according to CD3^+^ T cells at time of transplantation. Those with CD3^+^ T cells >6% presented an inferior rejection-free graft survival (at 3 months 43% vs. 100% in those with CD3^+^ =<6%; Log-rank *p* = 0.037), and a 15 times superior risk for an acute rejection during the first year (HR 14.9 [95% CI 2.4–92.4]; *p* = 0.04) (Figure 6B).

## Discussion

The work herein presented aimed at exploring the immune profiling of peripheral blood mononuclear cells and their correlation with acute rejection episodes in kidney-pancreas transplant recipients. Though an exploratory analysis, we were able to identify that during acute rejection episodes, CD4^+^ and CD8^+^ T cells, as well as CD19^+^ B cells, were increased compared to those without rejection. Moreover, we were able to identify that patients who developed an early acute rejection episode had higher T cells (either CD3^+^, CD4^+^, and CD8^+^) at the time of transplantation compared to the rest.

The finding of a positive correlation between the presence of an increased number of T and B cells during pancreas acute rejection episodes was somewhat expected, since it translates a normal immune response during inflammatory conditions, increasing both T and B cell trafficking as a response to local cytokine release. This may explain the increase in CD8^+^ naïve T cells, but not in other activation or differentiation subsets, such as effector memory T cells. This discordance between the activation and clonality of both T cells (20) and B cells (19) in peripheral blood when compared to those infiltrating the graft has been described in kidney transplantation, and may partially explain the limited ability of these cell subsets to discriminate between those with and without pancreas acute rejection, and halt its use as biomarker for acute rejection.

T-cell depleting agents have been widely used as induction therapy in pancreas transplantation in most centers worldwide (38) leading to a reduction in the incidence of acute rejection. Despite the use of thymoglobulin (a T-cell depleting polyclonal antibodies) as induction therapy, we identified that during early pancreas acute rejection episodes both T and B cell subsets presented a good capacity to discriminate between those with BPAR and those without rejection. There are some cell subsets that have been reported to be resistant depletion by T-cell depleting agents. Mouse anti-thymocyte globulin (mATG) preferential depletes naïve T cells, resulting in an increased ratio of regulatory and memory T cells within 1 day after mATG administration (39). In a mouse model of kidney transplant, ATG was effective in depleting T cells, but favored the expansion of T follicular helper cells following depletion. Treatment with ATG also increased germinal center B cells and lead to higher titers of antigen-specific antibodies compared to controls (40). Though a small percentage of those who receive a *de novo* simultaneous kidney-pancreas transplant is sensitized (20%), pre-transplant work up is based only on humoral response (anti-HLA antibodies), and no functional cellular analysis was performed to determine the presence of donor-specific memory T cells at time of transplantation.

T cells subpopulations have been described to correlate with the risk of acute rejection after kidney transplantation (2). In our study we identified those patients with pre-transplant CD3^+^ T cells >6% presented an increased the risk for pancreas acute rejection during the first year up to 15 times. The small sample size halts the extrapolation of these data to clinical practice. These results concur with a recent study from Chellappa et al, which also identified that patients’ who develop acute rejection during the first year after transplantation present at the time of transplant an increase in activated CD3^+^ T cells, both CD4^+^ and CD8^+^ (38). As postulated previously, this might be correlated to the presence of donor-specific memory T cells prior to transplantation. Moreover, it must be taken into consideration that most pancreas transplant recipients had type 1 diabetes mellitus (T1D). T1D is an autoimmune disease that may relapse after pancreas transplantation, and the presence of islet specific T cells identified in peripheral blood during relapse in the pancreas graft. Yet considered in pancreas transplantation, may be a potential role autoimmunity triggering an alloimmune response. Pancreatic beta cells express major histocompatibility type II (MHC-II) and during inflammatory conditions may behave as antigen presenting cells (APC) (41). In mouse models of islet transplantation, activation of auto-reactive T cells leads to rejection of the islet graft mediated by alloreactivity (42). Hence, to which extent the herein identified increase in peripheral blood CD4^+^ memory T cells at time of transplantation may translate an increase in auto-reactive T cells and subsequent graft rejection remains to be addressed.

Another relevant finding in our study was the increase in CD19^+^ B cells in patients with acute rejection. The diagnosis of antibody-mediated rejection (ABMR) in pancreas transplantation, and according to the Banff classification (36), depends on the presence of characteristic histological lesions, presence of C4d staining, and circulating DSA. The later correlate not only with an increased risk for graft failure (33, 34), but their presence has also been associated with sub-clinical acute rejection episodes. In our study we have identified that during acute rejection episodes CD19^+^ B cells were increased, despite having only one case of biopsy-proven acute rejection. Nonetheless, up to 38% had DSA at the time of biopsy. In a series of pancreas graft performed per protocol biopsies, Uva et al identified that 54% of the patients did not present signs of acute rejection despite having circulating DSA (43). These results correlate with another recent study in which, exploring a gene set to evaluate the expression of ABMR in pancreas graft biopsy, there was no correlation between the presence of DSA and ABMR gene expression (44). Finally, we have recently reported that donor-derived cfDNA was increased in patients with DSA despite the absence of signs of ABMR in graft biopsy (45). Altogether, these studies highlight that histological ABMR may be underdiagnosed in pancreas transplantation, and is important to design larger studies in patients with pancreas ABMR aiming at exploring the molecular and genetic biomarkers, and more in depth functional analysis of peripheral blood mononuclear cells. NK cells, which were proportionally similar between those with and without pancreas acute rejection, have been described as key players in ABMR and chronic ABMR in kidney transplantation (46).

The authors would like to highlight some additional limitations to this study. The first and most relevant relies on the small single center cohort, which limits the validity of the results and their extrapolation to other populations. Despite the longitudinal design, pancreas transplantation is a minority procedure, with a median of 15 procedures/year performed at our center. This study was also not designed to perform a longitudinal evaluation of circulating leukocytes at different time-point of the post-transplant period, which may bias interpretation of subsets of populations in biopsies performed early after transplantation due to the use of induction therapy with T-cell depleting agent. Despite this limitation, on the longitudinal study only first SPK transplant recipients were included, and the observed correlation of T cells at transplantation with post-transplant outcomes is not biased by immunosuppression. Moreover, the high acute rejection rate observed in our population may be related to the fact that almost a third relied on clinical criteria, which may have led to an overdiagnosis and treatment. Finally, in biopsy-related samples, indication for biopsy was dependent on the attending physician criteria, which may differ from other centers practices.

In conclusion, to the authors’ knowledge this is the first study aiming at exploring immune cell profiling in kidney-pancreas transplant recipients and their correlation with pancreas graft acute rejection. These results pave the way towards more in depth studies that may further characterize these cellular populations and ultimately lead to the individualization of immunosuppression.

## Data Availability

The raw data supporting the conclusion of this article will be made available by the authors, without undue reservation.
