# Supplementary material for: Immune Profiling of Peripheral Blood Mononuclear Cells at Pancreas Acute Rejection Episodes in Kidney-Pancreas Transplant Recipients
Source: Transpl Int. 2022 Nov 18;35:10639. doi: 10.3389/ti.2022.10639 (PMC9715609; doi:10.3389/ti.2022.10639)
Supplement: Supplementary file 1 [file DataSheet1.PDF]

Immune profiling of peripheral blood mononuclear at pancreas acute rejection episodes in kidney-pancreas transplant recipients

SUPPLEMENARTY DIGITAL CONTENT

Table S1. Flow cytometry antibodies for lymphocyte characterization.

Table S2. Demographic, immunologic, and clinical parameters of Simultaneous Pancreas Kidney transplant recipients followed for 12months

Figure S1. Overview of the gating strategy for T and B cell subsets.

Table S1. Flow cytometry antibodies for lymphocyte characterization.

Marker	Dye	Cat. Nº	Clone	Company
IgD	FITC	11-9668-42	IA6-2	Invitrogen
CD16	FITC	561308	B73.1	BD Pharmigene
TCRγδ	FITC	11-09959-41	B1.1	Invitrogen
CD57	FITC	11-0577-42	TB01	Invitrogen
CD127	FITC	11-1278-42	EBicRDR5	Invitrogen
CD21	PE	12-0219-42	HB5	Invitrogen
CD5	PE	12-0059-42	UCHT2	eBioscience
CD56	PE	12-0566-42	TULY56	Invitrogen
TCRαβ	PE	12-9986-42	IP26	Invitrogen
CD28	PE	555729	CD28.2	BD Pharmigene
CCR7 (CD197)	PE	12-1979-42	3D12	Invitrogen
CD27	PerCP-eF710	46-0271-82	LG7F9	Invitrogen
CD3	PerCP-eF710	46-0037-42	OKT3	Invitrogen
IgG	PE-Texas Red	562538	G18-145	BD Bioscience
CD24	PE-Cy7	25-0247-42	eBioSN3	Invitrogen
B220	PE-Cy7	552772	RA3-6B2	BD Biosciences
CD8a	PE-Cy7	46-0087-42	SK1	Invitrogen
CD25	PE-Cy7	25-0257-42	CD25-4E3	Invitrogen
CD19	APC	17-0198-42	SJ25C1	Invitrogen
CD27	APC	17-0279-42	O323	Invitrogen
CD14	APC	345787	MφP9	BD Bioscience
CD45RO	APC	17-0457-42	UCHL1	Invitrogen
HLA-DR	APC	559866	G46-6	BD Pharmigene
CD62L	APC	17-0629-42	DREG-56	eBioscience
CD38	APC-Cy7	47-0389-42	HIT2	Invitrogen
CD19	APC-Cy7	47-0199-42	HIB19	Invitrogen
CD4	APC-Cy7	47-0048-42	OKT4	Invitrogen
CD69	APC-Cy7	560737	FN50	BD Pharmigene
IgM	V450	561286	G20-127	BD Bioscience
CD3	V450	48-0037-42	OKT3	Invitrogen
CD64	V450	561202	10.1	BD Bioscience
CD45RA	V450	48-0458-42	HI100	Invitrogen
CD45	V506	69-0459-42	HI30	Invitrogen
Aqua live-dead	Pacific orange	L34957	---	Thermo Fisher

Table S2. Demographic, immunologic, and clinical parameters of Pancreas transplant recipients with biopsy performed during the first 3 months

		**Pancreas Rejection at 3months**		
	**Overall** (n=15)	**No Rejection** (n=10)	**Acute Rejection** (n=5)	***p***
**Recipient**				
Age (years)	40 ± 9	42 ± 9	36 ± 7	.18
Gender (male;%)	47%	50%	40%	.57
Type of DM (T1D; %)	87%	80%	100%	.43
Type of dialysis				.68
Pre-emptive (%)	7%	10%	0%	
Hemodialysis	80%	80%	80%	
Peritoneal Dialysis	13%	10%	20%	
Time on dialysis (months)	15 [8-12]	12 [8-18]	14 [13-14]	.48
**Donor/Transplant**				
Age (years)	39 ± 10	37 ± 12	44 ± 8	.44
Gender (male; %)	40%	50%	20%	.29
CIT (hours)	8 [7-9]	8 [7-19]	8 [7-10]	1.0
**Immunological**				
cPRA (%)				.24
Class I	0 [0-0]	0 [0-0]	0 [0-0]	.59
Class II	0 [0-6]	0 [0-0]	0 [0-6]	.67
Total	0 [0-6]	0 [0-0]	0 [0-6]	.78
Pre-formed DSA (yes)	13%	10%	20%	.57
Sensitized pre-transplant[¥]	20%	20%	20%	.75
Total Mismatches	5 [4-6]	5 [4-6]	5 [4-5]	1.0
Mismatches AB	3 [3-4]	4 [2-4]	3 [3-3]	1.0
Mismatches DR	2 [2-2]	2 [1-2]	2 [2-2]	.80
Maintenance Immunosuppression				.56
PDN+TAC+MMF	73%	60%	100%	
PDN+TAC+SRL	7%	10%	0%	
TAC+MMF	20%	30%	0%	
**Acute rejection**				
Biopsy Histological classification				
No Rejection		N=10		
Indeterminate			n=2	
Acute Cellular grade 1			n=2	
Antibody mediated rejection			n=1	
Time to biopsy (months)	0.9 [0.7-1.5]	1.2 [0.8-1.5]	0.7 [0.6-1.3]	.12

¥ Sensititzation defined as the presence of pre-formed DSA and/or total cPRA >50%. DSA – donor specific antibodies; MMF – Mycophenolate Mofetil; PDN – Prednisone; SRL – Sirolimus; TAC – Tacrolimus. T1D – Type 1 Diabetes Mellitus.

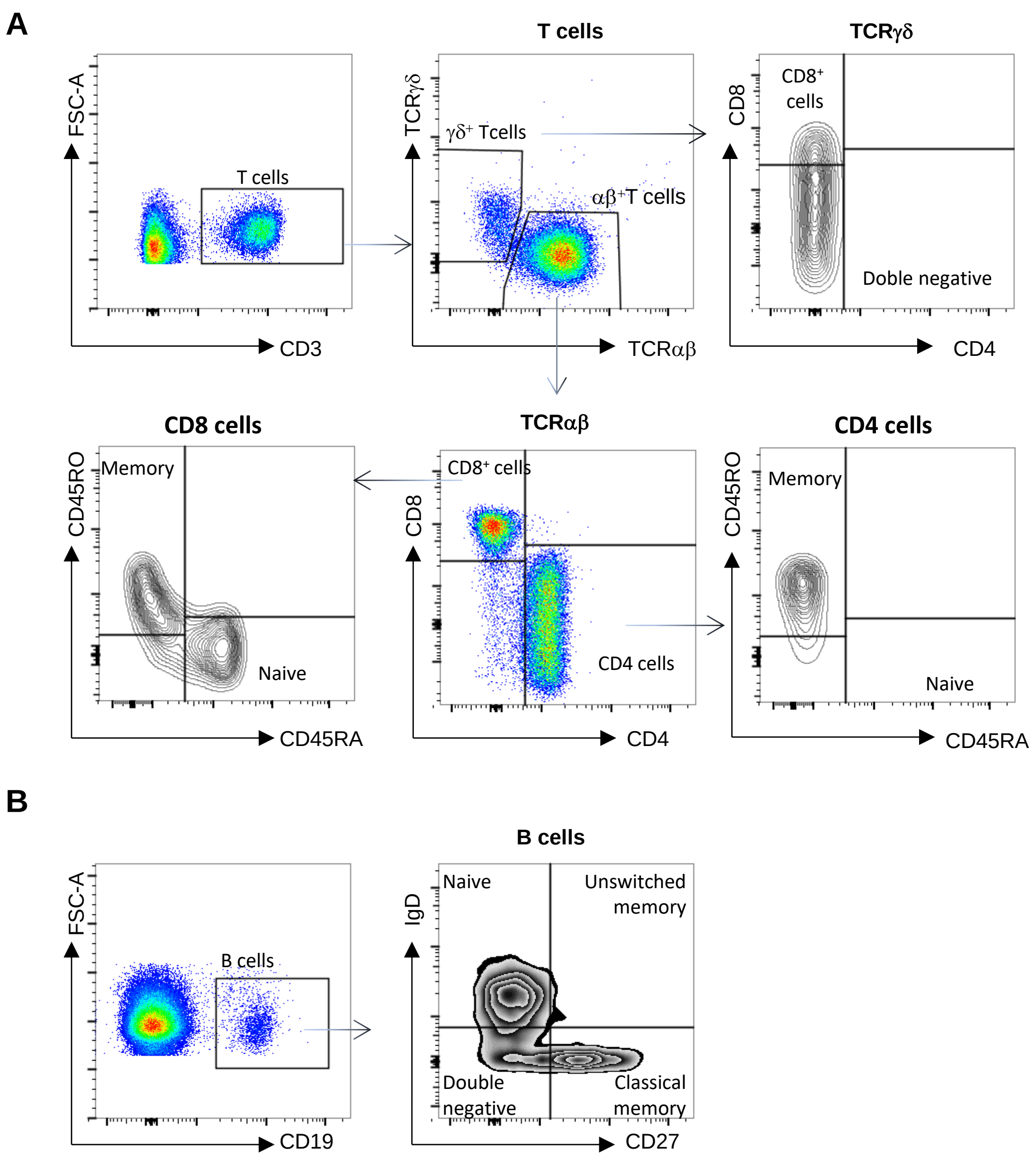

Figure S1. Overview of the gating strategy for T and B cell subsets. The data file of the stained peripheral blood mononuclear cells (PBMC) was analyzed. (**A**) Gating strategy for T cell subsets. Following gating strategy up to living cells; gating of CD3+ T cells (CD3 *vs* FSC-A); gating of $\alpha\beta^+$ T cells and $\gamma\delta^+$ T cells (TCR$\alpha\beta^+$ *vs* TCR$\gamma\delta^+$); and gating of $CD4^+$ and $CD8^+$ T cells for both T cell receptor subsets (CD4 *vs* CD8). CD8 and CD4 were classified as naïve or memory cells according CD45RA and CD45RO surface markers. (**B**) Gating strategy for B cell subset. Following gating strategy up to living cells; gating of CD19+ T cells (CD19 *vs* FSC-A). CD19 were classified as naïve, unswitched memory, classical memory, and double negative B cells according IgD and CD27 surface markers.
